# Hepatotoxicity Evaluation of Levornidazole and Its Three Main Impurities: Based on Structure–Toxicity Classification Prediction Combined with Zebrafish Toxicity Assessment

**DOI:** 10.3390/molecules30050995

**Published:** 2025-02-21

**Authors:** Ting Liu, Song Yuan, Luyong Zhang, Dousheng Zhang

**Affiliations:** 1 Institute for Food Control, National Institutes for Food and Drug Control, Beijing 102629, China; lutyliu@126.com (T.L.); zhangluyong301@126.com (L.Z.); 2 Institute for Drug Control, National Institutes for Food and Drug Control, Beijing 102629, China; yuansong@nifdc.org.cn

**Keywords:** levornidazole, impurities, hepatotoxicity, classification prediction model, zebrafish

## Abstract

Levornidazole, a nitroimidazole compound, has been linked to hepatotoxic adverse effects in clinical settings. However, the hepatotoxicity of levornidazole and its impurities has not been fully elucidated. This study aimed to predict and evaluate the potential hepatotoxicity of levornidazole, and elucidate the underlying mechanisms of action. Computational models based on support vector machines (SVM) and artificial neural networks (ANN) predicted that levornidazole, ornidazole, and impurity II exhibited hepatotoxic effects. The hepatotoxicity of levornidazole and impurity II was confirmed using a zebrafish toxicity study, with impurity II demonstrating hepatotoxicity at lower doses. Molecular structure analysis revealed that the electronegativity of the side-chain groups and the molecular polarity structure were correlated with the degree of hepatotoxicity. The toxic response was primarily associated with specific structural domains of the molecule, including the 2-methyl-5-nitro-1H-imiddaster-1-yl structure and the substituent groups of 1-chloro and 2(S)-2-methyloxirane. Transcriptome sequencing analysis indicated that levornidazole and impurity II affect multiple metabolic processes in the liver, including glucose, lipid, protein, hormone, and drug metabolism. These findings highlight the potential hepatotoxic risks associated with levomeprazole and its impurities, emphasizing the importance of further investigation and regulatory attention to ensure patient safety.

## 1. Introduction

Drug reevaluation involves a thorough and comprehensive study to assess the quality and safety of drugs already available on the market. By continuously monitoring clinical safety and efficacy, it becomes evident that serious adverse events in the clinic due to the potential drug toxicity and impurities pose a significant risk to the quality and clinical safety of drugs [1]. The recent market recall or withdrawal of the commonly used medications, such as ranitidine and valsartan, has been prompted by safety concerns related to the carcinogenic impurity N-nitroso dimethylamine (NDMA) in various regions, including the United States, Canada, the European Union and China, underscores the critical need for conducting comprehensive and objective evaluations of impurity toxicity in drugs [2,3].

Levornidazole is a newly developed nitroimidazole compound characterized by the chemical formula C_7_H_20_ClN_3_O_3._ In comparison to its predecessors, metronidazole and tinidazole, it demonstrates superior efficacy against anaerobic microorganisms, including *Bacteroides fragilis*, *Parabacteroides distasonis*, *Enterococcus*, *Streptococcus*, *Helicobacter pylori*, and *Porphyromonas gingivalis* [4]. However, nitroimidazole derivatives may cause hepatotoxic damage resembling acute cholestatic hepatitis [5]. There is a conspicuous scarcity of studies investigating the hepatotoxicity of levornidazole and its related impurities, which include ornidazole, levornidazole impurities II (IMP-II), and levornidazole impurities III (IMP-III). This gap highlights an urgent need for research to understand and mitigate the potential liver toxicity risks associated with these compounds.

The absence of established safety data for levornidazole and its impurities presents a significant risk to patients, potentially curtailing the drug’s clinical utilization [6,7]. Predictive methodologies such as structure–toxicity relationship (STR) based classification and quantitative structure–toxicity relationship (QSTR) models play a pivotal role in the prediction of drug toxicity. These models significantly enhanced detection rates for both known and previously unidentified toxicities [8,9,10,11,12,13,14]. Additionally, zebrafish are widely used in drug toxicity research due to their genetic similarity to humans, and the benefits of intuition as well as brief experimental period, which can elaborate a detailed examination of the drug toxicity and occurrence mechanism at both phenotypic and molecular levels [15,16,17,18,19].

In this study, we evaluated the hepatotoxic effects of levornidazole and its three primary impurities by constructing a toxicity prediction model and utilizing a zebrafish toxicity assessment. Our approach involved analyzing the correlation between the structural properties of the compounds and their potential hepatotoxicity effects. Furthermore, we sought to unravel the mechanism underlying their hepatotoxicity. The findings of this research not only contribute to improving the evaluation of levornidazole’s quality and safety profile in a clinical setting but can also be applied to other drugs and their impurities, enhancing the ability to identify and mitigate potential safety risks before ADR.

## 2. Results

### 2.1. Hepatotoxicity Prediction Modeling

The structures were converted to the SMILES code of 247 selected compounds and fed into the ADMET Predictor 9.5™. A total of 269 molecular descriptors were obtained by using the property prediction module, which included 8 simple constitutional descriptors, 6 topological indices, 57 atom-type electropropological state indices, 55 charge-based descriptors, 15 hydrogen bonding descriptors, 11 molecular ionization descriptors, and 58 functional groups. The initial exploration of modeling is significant in most cases [20], and then a well model was established. Therefore, these strategies were adopted to explore the indispensable conditions of a preliminary robust model and focused on the determination of modeling methods. While the classification value for hepatotoxicity was designated as the dependent variable, the 269 molecular descriptors were specified as the independent variables in the modeler module. The modeling exploration includes descriptor sensitivity, train/test set selection, model types, model performance, model selection, and so on. Among them, model selection was an interactive analysis, and several statistical metrics were given after each model.

We use ANN and SVM algorithms for modeling based on the framework. Then, we optimize the model using the Model Performance Grid. After repeated modeling, we optimized the chosen method’s specific parameters step by step. We used the ANN [21] and SVM [22] models to build a successful prediction model for hepatotoxicity. We chose ANN [21] as the modeling method. TLA [23] analyzed the descriptor sensitivities, while KSOP [24] handled the test set selection. For SVM, we used GA [25] to analyze sensitivities and KSOP [24] again for the test set selection. The optimal modeling parameters are in Table 1.

After conducting five rounds of testing and training, we showed the results in Table 2. For the ANN-1 classification model, we found the following metrics: Sensitivity: 0.84, Specificity: 0.81, False Positive Rate: 0.18, Youden’s Index: 0.65, Minimum Confidence: 0.55; All these indices surpass the performance metrics of the SVM model.

**Figure 1:**
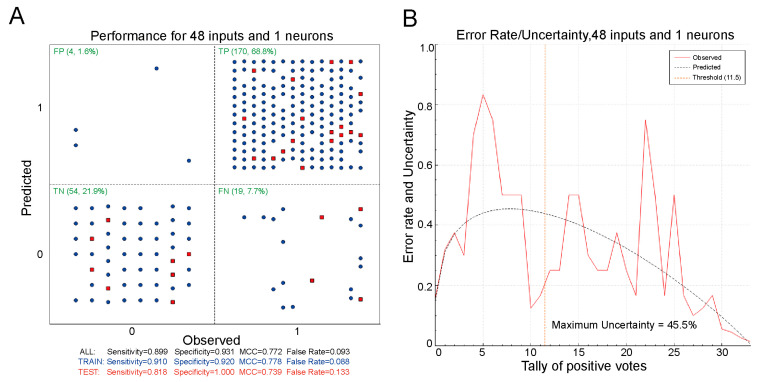
ANN1 Confidence Analysis Plots. (**A**) Scatter (48 × 1) plot, a graphical two-way truth table. Observed positives are in the right cells. Observed negatives are in the left cells. Predicted negatives are in the upper cells, while predicted positives are in the lower cells, each class—false negatives (FN), true positives (TP), true negatives (TN), and false positives (FP) show its number and fraction in the top left corner of the respective quadrants, training set points are represented in blue, test set points are highlighted in red; (**B**) Youden Plot, a plot of observed and predicted error rates as a function of the degree of concordance between the individual models that make up the ensemble, the plot shows the maximum uncertainty at the bottom, the predicted uncertainty profile appears as a black dashed line, the red line shows the observed profile, the yellow line represents the threshold profile, errors on the left side of the threshold show false negatives, errors on the right side indicate false positives.

The Scatter (48 × 1) plots showed the following results: False Positive (FP): 1.6%, True Positive (TP): 68.8%, True Negative (TN): 21.9%, False Negative (FN): 21.9%. See Figure 1A for details. With a lower FP of 1.6%, the ANN-1 model has good sensitive, accurate, and predictive performance in classifying hepatotoxicity. Furthermore, Figure 1B illustrates Confidence Analysis, where the maximum uncertainty of predicted errors is 45.5%, which is less than 50%, and the minimum confidence is 0.55, greater than 0.5. These results show that the dataset distribution is balanced, which means it fits well with a single, unified distribution. Based on these findings, we selected ANN-1 as the ultimate prediction model.

### 2.2. Prediction of Compound Hepatotoxicity

The ANN-1 model predicted potential hepatotoxicity for levornidazole, ornidazole, IMP-II, and IMP-III. The results are in Table 3. The chance of hepatotoxicity was 99% for both levornidazole and ornidazole. In contrast, IMP-II had a moderate probability of 56%. IMP-III was found to have a low potential for hepatotoxicity. Its likelihood is only 54%.

Levornidazole and three main impurities were also tested for possible hepatotoxicity using an online prediction tool, and the outcomes were compared to the newly constructed ANN1 model. In contrast to the online predictions, which all indicated that impurity II had potential hepatotoxicity (Table 4), the newly constructed ANN1 model predicted that impurity III did not contain potential hepatotoxicity (54% likelihood).

### 2.3. The Impact of Structural Characterization on Hepatotoxicity

**Figure 2:**
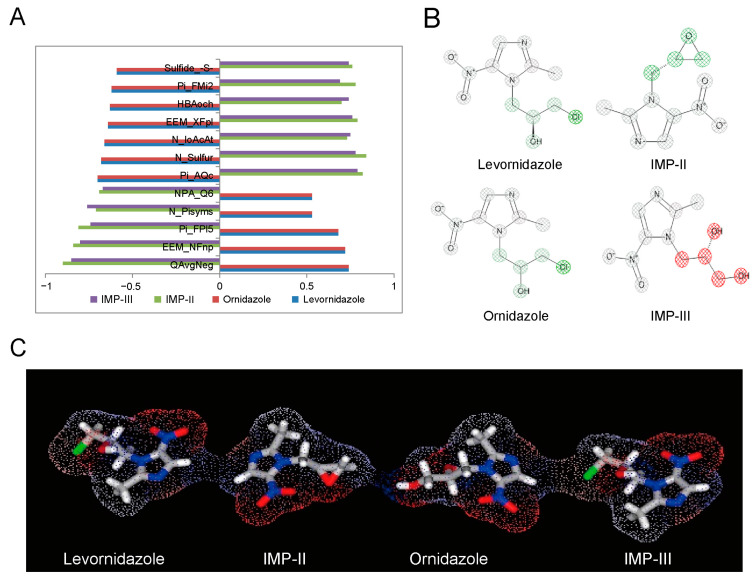
Correlation between molecular structural features of compounds and hepatotoxicity. (**A**) Distribution of characteristic descriptors associated with hepatotoxicity; (**B**) Distribution of characteristic domains associated with hepatotoxicity (red: negative correlation, green: positive correlation); (**C**) Molecular charge density distribution (red: negative charge, blue: positive charge).

To further characterize the hepatotoxic properties of levornidazole and its impurities, we analyzed how the structure of these compounds correlates with hepatotoxicity. Utilizing ADMET Predictor™ 9.5 software, we evaluated [30] molecular descriptors employed in the ANN model to isolate descriptors significantly associated with hepatotoxicity. As shown in Figure 2A and Supplementary Table S1, the potential hepatotoxicity of levornidazole and its three major impurities was closely associated with 11 characteristic molecular descriptors. These include six charge-based descriptors, one functional group descriptor, one hydrogen bonding descriptor, two molecular ionization descriptors, and two simple conformational descriptors.

Among them, descriptors like QavgNeg, EEM_NFnp, Pi_FPl5, N_Pisyms, and NPA_Q6 were positively associated with the hepatotoxicity of levornidazole and ornidazole, while descriptors such as Pi_Aqc, N_Sulfur, HBAoch, N_IoAcAt, EEM_XFpl, Pi_FMi2, and Sulfide_-S- appeared to mitigate the risk. This resulted in a contrasting impact on the potential liver toxicity of IMP-II and IMP-III, suggesting significant structural dissimilarities between the compounds, particularly in their substituent variations.

Moreover, the substituent status of the side-chain substituents, charge distribution, molecular polarity, and spatial topology collectively contribute to the hepatotoxicity profile of the compound molecules. Compounds with higher electronegativity exhibit greater hepatotoxicity, while those with lower polarity also demonstrate increased hepatotoxicity. The sensitivity analysis focused on the molecular structure hepatotoxicity revealed that the presence of the 2-methyl-5-nitro-1H-imidazole-1-yl structure had an impact on hepatotoxicity. Substitutions such as 1-chloro and 2(S)-2-methyloxirane were found to have a positive effect on hepatotoxicity. Interestingly, in IMP-III, the substitution of (s)-propane-1,2-diol had a negative effect on hepatotoxicity (Figure 2B).

The charge distribution patterns in these compounds, seen in Figure 2C, show that the 2-methyl-5-nitro-1H-imidazole-1-yl structure strongly influences positive electronegativity. The 2(S)-2-methyloxirane and 1-chloro substituents also play a role, but their impact is smaller. On the other hand, (s)-propane-1,2-diol raises electronegativity less than the two substituents mentioned. However, it significantly boosts the molecule’s polarity. These insights show that the 2-methyl-5-nitro-1H-imidazole-1-yl structure boosts hepatotoxicity. It does this by enhancing its positive electropositive property. Also, the 2(S)-2-methyloxirane and 1-chloro substituents raise electronegativity. Together, these factors increase hepatotoxicity. A higher molecular polarity, like that of (s)-propane-1,2-diol, is linked to lower hepatotoxicity. This provides a protective effect.

### 2.4. MNLC and LC10 of Compounds

**Figure 3:**
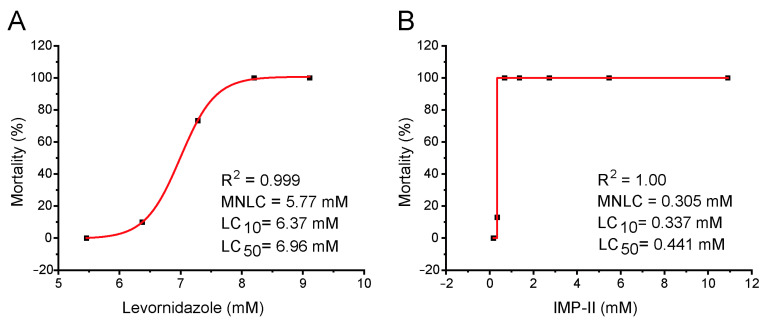
Compound-induced dose-dependent zebrafish mortality. (**A**) Levornidazole; (**B**) IMP-II. Treatment: from 0 to 48 h post drug treatment by 6-well plates (n = 30).

Our study examining the relationship between the concentration of target compounds and lethality found that none of the zebrafish survived when the concentration of levornidazole exceeded 6.37 mM or when the concentration of IMP-II exceeded 0.37 mM. We averaged the results from three experiments and derived the optimal concentration-lethal curves, which are depicted in Figure 3 [17,31]. The values for the Minimum Notable Lethal Concentration (MNLC), the lethal concentration for 10% mortality (LC10), and the lethal concentration for 50% mortality (LC50) were fitted from these curves. For levornidazole, the MNLC, LC10, and LC50 values were 5.77 mM, 6.37 mM, and 6.957 mM, respectively, with the LC50 confidence interval ranging from 6.774 mM to 7.139 mM. For IMP-II, the MNLC, LC10, and LC50 values were 0.305 mM, 0.337 mM, and 0.441 mM, respectively, and the LC50 confidence interval ranged from 0.386 mM to 0.508 mM.

### 2.5. Qualitative Assessment of Hepatotoxicity

**Figure 4:**
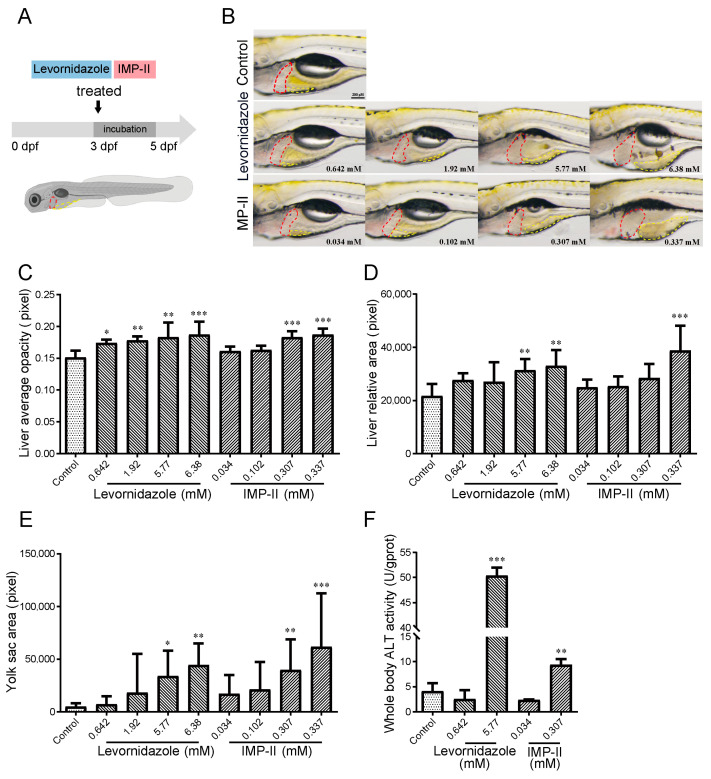
Observation of larval zebrafish hepatotoxicity phenotype after treatment with levornidazole and IMP-II for 48 h. (**A**) Schematic diagram of the zebrafish treatment stage. (**B**) Representative images displaying morphological alterations in the liver of zebrafish exposed to levornidazole and IMP-II. The liver (red dashed line) and yolk sac (yellow dashed line) were selected for quantification. Images of zebrafish are shown at 8× magnification with a scale bar of 200 µm. (**C**) Quantification of liver opacity following exposure to levornidazole and IMP-II. (**D**) Quantification of liver area following exposure to levornidazole and IMP-II. (**E**) Quantification of yolk sac area following exposure to levornidazole and IMP-II. (**F**) Quantification in ALT activity level following exposure to levornidazole and IMP-II. All data are presented as mean ± standard error of the mean (S.E.M). Asterisks indicate significant differences between the control and exposure groups: * *p* < 0.05, ** *p* < 0.01 and *** *p* < 0.001 compared to the control.

The effect of hepatotoxicity on zebrafish was evaluated through the analysis of liver morphology following treatment with compounds (Figure 4). Manifestations of hepatotoxicity in zebrafish larvae were marked by an enlarged liver area, liver opacity, and delayed yolk absorption. At the endpoint of the experiment (5 dpf), as shown in Figure 4B, larvae from the control group exhibited transparent hepatic tissue with discernible circulation of blood cells and fully absorbed yolk sacs. In stark contrast, the larval liver in the levornidazole and IMP-II treatment groups exhibited non-transparent from brown to dark. A noticeable reduction, if not a complete absence, of hepatic blood flow in a dose-dependent manner with levornidazole-treated doses ranging from 0.642 to 6.38 mM, and IMP-II-treated doses ranging from 0.307 to 0.337 mM (Figure 4C). Hepatomegaly was also induced at levornidazole concentrations of 5.77 mM and IMP-II concentrations of 0.337 mM (Figure 4D). Compared to the control, levamisole and IMP-II both led to delayed yolk absorption in a dose-dependent manner (Figure 4E). Additionally, the levels of ALT in zebrafish homogenate supernatant were significantly increased at the concentration of levornidazole (5.77 mM) and IMP-II (0.337 mM) (Figure 4F). No evidence of cardiotoxicity, such as arrhythmia, pericardial edema, or venous congestion, was observed in zebrafish following treatment with levornidazole and IMP-II. However, delayed development of the swim bladder was noted, indicating that levornidazole and IMP-II may affect swim bladder formation in zebrafish. This finding warrants further investigation. In summary, the study established the hepatotoxicity of both levornidazole and IMP-II, with IMP-II exhibiting hepatotoxic effects at lower doses compared to levornidazole.

### 2.6. Histopathology and Ultrastructure Changes

**Figure 5:**
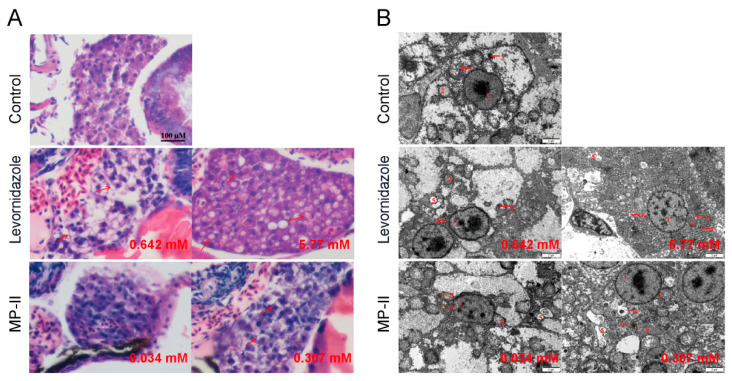
Histopathology and ultrastructure of larval zebrafish liver after treatment with levornidazole and IMP-II for 48 h. (**A**) Morphology of liver pathological sections (HE staining, 400× Scale bar is 100 µm). Liver vacuolar degeneration is pointed out by red arrows. (**B**) Hepatocytic ultrastructure by transmission electron microscopy. 1. cell nuclei; 2. mitochondria; 3. lysosomes; 4. endoplasmic reticulum; 5. lipid droplets; 6. myeloid vesicles.

To deepen our understanding of the hepatotoxic effects of the compounds, we investigated the histopathology and ultrastructure of the zebrafish liver. A histopathological evaluation of levornidazole and IMP-II induced hepatotoxicity was evaluated by hematoxylin and eosin (H&E) staining. The hepatocytes of zebrafish in the control group demonstrated well-preserved structural integrity and uniformity in cell morphology, with round, centrally situated nuclei within cells that were tightly and orderly arranged. However, exposure to both levornidazole and IMP-II led to typical manifestations of liver degeneration. As depicted in Figure 5A, hepatocytes from zebrafish treated with 0.642 mM of levornidazole exhibited vacuolized degeneration of hepatocytes. A severe degeneration was evident at a higher concentration of 5.77 mM, marked by hepatomegaly, with blunt rounded edges, enlarged hepatocytes, vacuolized degeneration, and cytoplasmic fading. The group exposed to a concentration of IMP-II at 0.034 mM showed no significant abnormalities in liver structure, similar to the control group. Notably, the group exposed to a concentration of 0.307 mM exhibited vacuolized degeneration of hepatocytes. The effects of the compounds on the ultrastructure of the zebrafish liver were further investigated. Transmission electron microscopy results revealed that the membranes and organelles of zebrafish hepatocytes in the control group were structurally integral. The nuclear envelope was sharply contoured with conspicuous perinuclear spaces, prominent nucleoli, and mitochondria ranging from regular rounded to oval shapes. The endoplasmic reticulum was neatly arrayed in parallel lines resembling flattened sacs. Contrastingly, in the group exposed to 0.642 mM levornidazole, the presence of lipid droplets was observed. At the higher levornidazole dose of 5.77 mM, cellular swelling was observed. The development of the perinuclear gap in the nucleus was hindered, but there was an increase in the number of mitochondria alongside a rise in the quantity of swelling, lysosomes, and myeloid vesicles. In the case of IMP-II, lipid droplets appeared in the 0.034 mM concentration group, and both lipid droplets and lysosomes appeared in the 0.307 mM concentration group. Additionally, the number of myeloid vesicles increased in both groups (Figure 5B).

### 2.7. Hepatotoxicity Mechanism of Compounds

**Figure 6:**
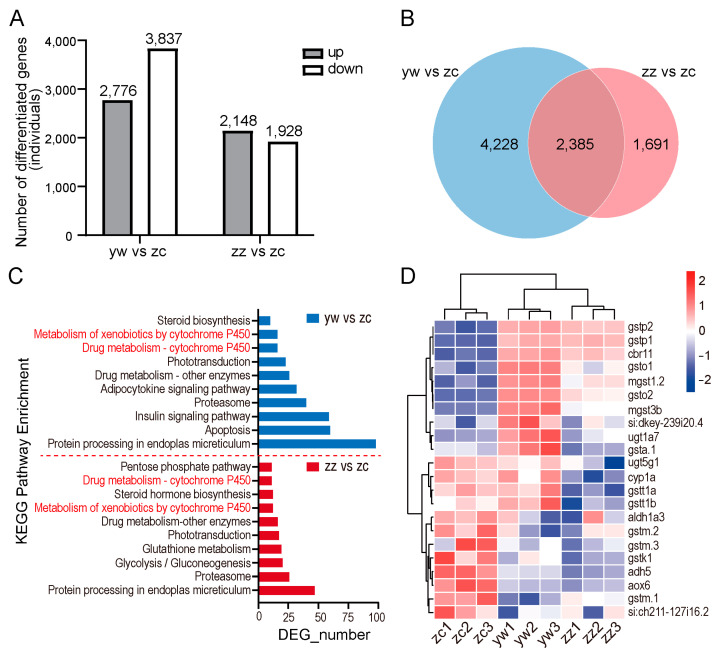
Transcriptome analysis of zebrafish after treatment by compounds. (**A**) Differentially expressed mRNAs (*p* < 0.05 and |log2FC| ≥ log22.0). (**B**) yw or zz co-regulated genes. (**C**) Gene counts in different organismal systems obtained by KEGG classification. (**D**) Heatmap showing the differential mRNA expression on the cytochrome P450 pathway. The zc represents the control group, the yw represents the group treated with 5.77 mM levornidazole, and the zz represents the group treated with 0.307 mM IMP-II.

To elucidate the mechanism implicated in compound-induced hepatotoxicity in zebrafish, a transcriptome analysis was conducted on zebrafish treated with levornidazole and IMP-II. Comparative analysis revealed that in the 5.77 mM levonidazole group, 2776 genes were upregulated and 3837 genes were downregulated compared to the control group. Similarly, in the 0.307 mM IMP-II group, result in 2148 genes upregulated and 1928 genes were downregulated (Figure 6A). Further analysis identified genes that were uniquely or commonly regulated by either treatment: a total of 2385 differential genes showed differential expression both in the comparison of control with levornidazole and control with IMP-II groups (Figure 6B). Distinct differential expressions were observed, with a total of 4228 expressed genes regulated by levornidazole and 1691 by IMP-II. KEGG analysis results indicated that both substances affect the metabolism of substances by the liver, such as sugars, fats, proteins, and hormones through pathways including glycolysis/gluconeogenesis, the insulin signaling pathway, the adipocytokine signaling pathway, protein processing in the endoplasmic reticulum, and steroid biosynthesis. Notably, disruptions in protein processing within the endoplasmic reticulum—an essential cellular function involving protein folding, modification, and transport—could lead to endoplasmic reticulum stress and possible cell apoptosis, which plays a significant role in the onset of liver diseases (Figure 6C). Moreover, levornidazole and IMP-II were found to disrupt drug metabolism and the xenobiotics mediated by cytochrome P450, resulting in abnormal upregulation of specific genes such as gstp1, gstp2, ugt1a7, and gsto, and abnormal downregulation of genes such as adh5, aldh1a3, gstk1, and aox6 (Figure 6D). These alterations hint at potential disturbances in detoxification processes and might be essential contributors to the hepatotoxicity observed in the zebrafish model.

## 3. Discussion

The latest advancements in liver toxicity prediction combine cutting-edge technologies with a deeper understanding of biological mechanisms [32,33]. In our study, we combined model accuracy, toxicity assessment, and mechanistic studies, and looked at the quality control and possible clinical risks of levornidazole. Finally, we not only established a new ANN model to predict the potential hepatotoxicity of target compounds rapidly, but also established zebrafish experiments in combination with histological experimental techniques to assess the degree of hepatotoxicity and elucidate the mechanism of hepatotoxicity.

Our investigations revealed for the first time that levornidazole and IMP-II are potentially hepatotoxic, with IMP-II exhibiting toxicity at lower doses, and that the presence of IMP-II in levornidazole formulations may pose a higher risk of liver injury. Our findings indicate that the hepatotoxicity classification prediction model developed using ANN [21] outperformed the model created with Support Vector Machines SVM [22] in terms of accuracy, sensitivity, and reliability (see Table 1, Figure 1A). This superior performance may be attributed to the fact that hierarchical neural networks are better suited for deep learning. Using the TLA method determines which variable selection method will be used to order the descriptors for inclusion in the model to improve descriptor selection matching effectively [23]. The dataset distribution fits well by a single, unified distribution (see Figure 1B). The analysis of specific structural features associated with hepatotoxicity, including the 2-methyl-5-nitro-1H-imidazole-1-yl structure and the substituents 1-chloro and 2(S)-2-methyl oxirane, reveals that factors such as electronegativity and the molecular polarity of side-chain groups correlate with hepatotoxicity. This understanding contributes to a theoretical framework for the mechanisms of hepatotoxicity. We also found that while online predictions indicated that impurity III might be hepatotoxic (see Table 4), our model revealed only a 54% likelihood of hepatotoxicity. This aligns with the results from our zebrafish experiments. Therefore, we exercise caution when using these online predictions in our study.

Furthermore, the zebrafish transcriptome analysis highlighting the effects on various metabolic pathways in the liver emphasizes the complexity of drug-induced liver injury and the necessity of considering both structural and functional elements in toxicity evaluations [34]. First, we demonstrate that research validated computational predictions using the zebrafish model, showing a high level of consistency in the results. Additionally, RNA-Seq was utilized to identify genes and pathways associated with increased liver toxicity, the use of RNA-Seq technology allows for the observation of dynamic changes in gene expression across various tissues. The investigation focused on the potential mechanisms involved in levornidazole and IMP-II. The findings revealed an impact on the liver’s detoxification processes, particularly affecting pathways such as Drug Metabolism—Cytochrome and Metabolism of Xenobiotics by Cytochrome. This resulted in an abnormal upregulation of genes like gstp1, gstp2, ugt1a7, and gsto, alongside an abnormal downregulation of genes such as adh5, aldh1a3, and gstk1. By analyzing gene expression differentials at the RNA level under different influences, we can link specific differentially expressed genes to distinct biological functions. This approach facilitates the exploration of early damage biomarkers and molecular regulatory mechanisms. Further exploration of these mechanisms will take place in subsequent studies. The ANN1 models have shown high sensitivity and specificity in predicting hepatotoxicity; however, their performance can be affected by the quality and quantity of the training data. Additionally, improving the interpretability of these models could provide clearer insights into the structural features that contribute to toxicity. Although the zebrafish model is a valuable tool for toxicity screening [17,18,19], it has limitations in terms of fully replicating human liver physiology and pathology [35]. Future research should focus on validating the findings using more diverse and larger datasets to strengthen the reliability of the prediction models. Additionally, investigating the hepatotoxicity of other impurities and metabolites of levornidazole will contribute to a more comprehensive understanding of the drug’s safety profile.

## 4. Materials and Methods

### 4.1. Hepatotoxicity Predictions

#### 4.1.1. Data Preparation

The dataset utilized for constructing the model was obtained from DrugBank (https://www.drugbank.ca/; accessed on 3 August 2024) [36] and SIDER.4.1 (http://sideeffects.embl.de/; accessed on 3 August 2024) [37,38] by containing the subject term hepatotoxicity or liver injury, resulting in a collection of 1418 chemical structures associated with hepatotoxicity. Subsequently, the relevant data for these chemicals were downloaded and inputted into the ADMET Predictor™ 9.5 software (version 9.5.0.16, simulations plus, Inc., Lancaster, CA, USA) [39], with specific parameters set as follows: (1) Using the imidazole structure as the parent ring. (2) The molecular selection range defined as 150 < MWt < 550, −3.0 < S + logP < 7.5 and 1< HBA < 12. (3) Hepatotoxicity or non-hepatotoxicity reported in the literature was used as the endpoint for determination, including data on liver injury from toxicity-related pathways such as metabolic enzymes, mitochondrial oxidative stress, cell proliferation and apoptosis, and immune-related pathways. (4) Exclusion of non-organic compounds, structurally similar compounds with significant differences in activity and compounds with incomplete toxicity information. Subsequently, 247 compounds were obtained, comprising 189 hepatotoxic and 58 non-hepatotoxic compounds, with detailed information provided in Supplementary Table S2.

#### 4.1.2. Modeling Protocol

**Figure 7:**
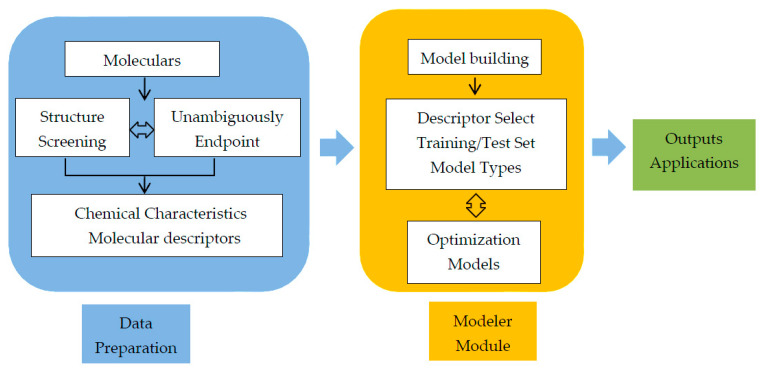
Framework for the classification prediction model of hepatotoxicity.

ADMET Predictor 9.5™ was used for the purpose of classification prediction modeling. Through the interactive interface, the property prediction and modeler module are used to automatically calculate and match the best model. Molecular descriptors were calculated and extracted from structural information of compounds and activity attributes in property prediction module. The descriptor selection, dataset splitting, model type and performance, model selection and assessment were performed in modeler module. The detailed modeling framework is shown in Figure 7.

#### 4.1.3. Descriptor Selection

The filtering of descriptors was performed in two stages using the ADMET Predictor 9.5™. The descriptor number reduction was set using the following criteria, including the minimal coefficient of variation set at <1%, the minimum representation set at 4, and the maximum absolute correlation set at >98%. At the same time, sensitivity analysis was executed using the following mathematical methods including input gradient, truncated linear analysis (TLA) [23], iterative truncated linear analysis (ITLA) [14], and genetic algorithm (GA) [25].

#### 4.1.4. Test Set Selection

A total of 247 modeling molecules were automatically assigned and assembled into training pools (for ANN and SVM models, the training pool was further subdivided into a training set and a verification set, for MLR model, the training pool was a training set) and a test set using the Kohen Map algorithm (KSOP) [24], K-Means Clustering [24], Random, and Stratified sampling [40], respectively. The set sizes of the training and test set compounds were distributed at a ratio of 5:1 (the minimum test set size is 10% of the input data).

#### 4.1.5. Model Types and Performance

Two machine-learning algorithms, ANN [21] and SVM [22], were used to generate the predictive models, using the descriptors and literature experimental data. The determination of the number of seeds involved using specific parameters of the algorithms and model fitting parameters. The model performances were demonstrated using the Model Performance Grid [41,42,43]. Sensitivity, Specificity, False Rate, Youden, and Min Confidence were selected as indicators to assess the accuracy, robustness, and explanatory capability of the constructed model. The parameters for each indicator were set as follows [44]: Sensitivity ≥ 0.9, Specificity ≥ 0.9, False Rate < 0.1, Youden ≥ 0.7, Min Confidence ≥ 0.5.

#### 4.1.6. Prediction of Compound Hepatotoxicity

Levornidazole and its impurities (ornidazole, IMP-II, IMP-III) were stored in 2D-SDF format, and a newly developed classification model (ANN) was used to predict hepatotoxicity. Additionally, the ADMET Predictor™ 9.5 software was used to analyze the characteristic descriptors (DSAs) and the structural sensitivities (SSAs).

### 4.2. Chemicals and Material

Levornidazole and IMP-II were purchased from the National Institutes for Food and Drug Control (NIFDC). Dimethylsulfoxide (DMSO) was obtained from Sigma. Levornidazole was dissolved in fish water. The IMP-II was dissolved in DMSO and different amounts of the master solutions were added directly to the fish water at designated testing concentrations, resulting in a final DMSO concentration of 0.5% (*v*/*v*). All solutions were freshly prepared before each experiment, and pH values were verified without any artificial adjustments.

Anhydrous ethanol (Lot# 20210107, Sinopharm Chemical Reagent Co., Ltd., Shanghai, China); Tissue cell fixation solution at 4% concentration (Lot# 20210828, Beijing Solarbio Science & Technology Co., Ltd., Beijing, China); Mayer hematoxylin stain solution (Lot# 20220120, Shanghai Yihe Biological Technology Co., Ltd.,Shanghai, China); Eosin (Lot# 20220120, Shanghai Yihe Biological Technology Co., Ltd., Shanghai, China); Glutaraldehyde (Lot# C10366283, Shanghai Macklin Biochemical Technology Co., Ltd.,Shanghai, China); Alanine aminotransferase Assay Kit (Lot# 20220517, Nanjing Jiancheng Bioengineering Institute, Nanjing, China).

### 4.3. Test Animals and Collection of Eggs

Zebrafish larvae were raised in accordance with established standard protocols as previously outlined [45]. AB strain wild-type zebrafish (*Danio rerio*) were maintained on a 14:10 h light:dark cycle at a temperature of 28.5 ± 0.5 °C. Zebrafish embryos were obtained through natural spawning. Briefly, adult males and females were housed separately for at least five days before spawning. Prior to egg collection, adult males and females were placed in breeding tanks with a male-to-female ratio of 3:2. The eggs were then collected one hour after the lights were turned on the following morning. All eggs were checked for fertilization and then combined in a single Petri dish containing fish water (0.2% Instant Ocean Salt in deionized water, with a pH of 6.9–7.2, conductivity of 450–550 mS/cm, and hardness of 53.7–71.6 mg/L CaCO3) until the start of the exposure. The protocols utilized for the experiments were approved by the Institutional Animal Care and Use Committee at Hunter Biotechnology, Inc. (Hangzhou, China). All animal experiments were conducted following the guidelines of the Institutional Animal Care and Use Committee (IACUC), and the procedures were approved by the IACUC (certification No. 001458), and the IACUC ethical approval number was IACUC-2022-4279-01.

### 4.4. Drug Treatment

Zebrafish embryos were reared until under standard conditions until 3 days postfertilization (dpf) before being used in experiments [46,47]. Three dpf larval zebrafish (n = 30) were stochastically distributed into 6-well plates containing 3 mL of fresh fish water and exposed to different concentrations of Levornidazole or IPM-II, respectively. All compounds were soluble in dimethyl sulfoxide (DMSO) and were diluted with fish water to achieve a final DMSO concentration of less than 1%. The fish water treatment group served as the negative control, while the 1% DMSO group acted as the vehicle control. The larvae were incubated at 28 °C, and dissolved oxygen concentration in each well was maintained above 80% throughout the experiments.

### 4.5. Zebrafish Lethality Ananlysis

To determine the maximum non-lethal dose (MNLC) 10% lethal dose (LD10) and 50% lethal dose (LD50) of the compounds, the larvae were treated with Levornidazole or IMP-II for 48 h. The assessment of mortality and toxicity was recorded every 24 h, and the dead zebrafish was defined as the absence of heartbeat under a dissecting stereomicroscope. The total number of dead zebrafish was applied to generate a lethality curve by plotting lethality (%) vs. concentration. Subsequently, the lethality curve, the MNLC, LC10 and LC50 were fitted and calculated using the OriginPro 8.0 (version 8.0, OriginLab Corpration, Northampton, MA, USA) statistical software.

### 4.6. Zebrafish Hepatotoxicity Assessment

Hepatotoxicity assessment was conducted in accordance with a previously established protocol with slight adjustments [48]. The concentrations of drugs used in the assessment of hepatotoxicity are outlined for lethality assays. Four concentrations, specifically LC10, MNLC, 1/3 MNLC, and 1/9 MNLC, were chosen based on the lethality curve and assessed for their hepatotoxic effects. The fish water treatment group was the control. At the end of treatment, 10 zebrafish from each group were randomly chosen for visual observation and image capture using a dissecting stereomicroscope. Data were collected and analyzed using NIS-Elements D 3.10 advanced image processing software to analyze hepatotoxicity based on three hepatotoxic phenotypic endpoints (liver size changes, liver degeneration, and yolk absorption delay). The results were presented as mean ± SE, and statistical analysis was conducted using SPSS26.0 software (version 26.0, IBM Corpration, Armonk, NY, USA), with *p* < 0.05 indicating statistically significant differences.

### 4.7. Liver Function Measurement

The liver function detection was performed using the GPT assay kit. Three dpf AB Zebrafish were treated with different concentrations of levornidazole (0.642 mM, 5.77 mM) or IMP-II (0.034 mM, 0.307 mM) in water, respectively. At the same time, a control group received an equal volume of water. Following a 48 h treatment at 28 °C under static conditions, a total of 30 larvae zebrafish were collected and homogenated. The ALT levels were determined using the GPT assay kit, following the instructions provided by the respective manufacturers. The experiment was independently repeated three times.

### 4.8. Histopathological Examination

For histological analysis, we followed previously described protocols with slight modifications [49,50]. The whole zebrafish were fixed with 4% PFA at 4 °C overnight. Following fixation, the zebrafish samples were washed with 70% ethanol, subsequent dehydrated in a series of increasing ethanol concentrations (70–100%), and were then embedded in paraffin. The paraffin-embedded tissues were sectioned serially at a thickness of 5 μm and stained with hematoxylin and eosin (H&E) for qualitative and quantitative histological analysis. The H&E-stained liver sections were observed using a light microscope (Olympus X71-F22PH, Tokyo, Japan) in a blinded manner. A comparative analysis was conducted between the liver sections of treated fish and those of control fish, encompassing both quantitative parameters (such as cell size and cell density) and qualitative observations (i.e., discernible changes in liver tissue). The experiment was independently repeated three times.

### 4.9. Transmission Electron Microscopy (TEM)

To assess the effects of compounds on the ultrastructure of the liver, the cellular organelle morphology of the liver was analyzed by transmission electron microscopy, following previously established methods with slight modifications. Zebrafish larvae samples were preserved in Karnovsky fixative (2% paraformaldehyde, 2.5% glutaraldehyde, 0.08 M Na cacodylate, pH 7.4, 0.25 mM calcium chloride, 0.5 mM magnesium chloride) at 4 °C overnight, and then embedded in propylene oxide and epoxy resin at 37 °C overnight. The embedded samples were sliced into ultrathin sections (60 nm), double-stained with 3% uranyl acetate and lead citrate, and subsequently observed using transmission electron microscopy. The experiment was independently repeated three times.

### 4.10. RNA-Sequencing Analysis

Zebrafish larvae were collected and cleaned with ultrapure water three times, followed by rapid freezing in liquid nitrogen and storage at −80 °C. Subsequently, the total RNA was extracted, and its integrity was evaluated using an Agilent 2100 Bioanalyzer. For the preparation of complementary DNA (cDNA) libraries, 3 μg of total RNA were captured by Dynabeads Oligo (dT) and fragmented to approximately 300 bp. Reverse transcription was performed by the Superscript III cDNA Synthesis Kit. Following library construction, PCR amplification was used for fragment enrichment, and the library was then selected based on a fragment size of 450 bp. Subsequently, the library was quality checked by Agilent 2100 Bioanalyzer, and the total concentration as well as the effective concentration of the library were determined. Based on the effective concentration and the required data amount, libraries containing different Index sequences were proportionally combined to differentiate the downstream data of each sample. The mixed libraries were uniformly diluted to 2 nM and denatured to form single-stranded libraries. Following RNA extraction, purification, and library construction, these libraries underwent double-end (paired-end, PE) sequencing using next-generation sequencing (NGS) technology on the Illumina sequencing platform. The experiment was independently repeated three times.

### 4.11. Statistical Analysis

Statistical analyses were performed with one-way ANOVA and Dunnett’s test using the SPSS 26.0 software (version 26.0, IBM Corpration, Armonk, NY, USA). All data were shown as the mean ± standard deviation (SD). All figures were generated by GraphPad Prism Software (version 8.3.0, GraphPad, Inc., San Diego, CA, USA).

## 5. Conclusions

This study combines computational predictions with zebrafish toxicity assessments and transcriptomics to provide a thorough evaluation of the hepatotoxicity of levornidazole and its impurities. The study provides valuable insights into the hepatotoxicity of levornidazole and its impurities. However, it does have some limitations, including challenges in the interpretability of the model and a lack of detailed explanations of the mechanisms underlying hepatotoxicity. In future, we plan to incorporate more in vitro and in vivo data, investigate additional impurities and metabolites, and conduct more in-depth mechanistic studies to further enhance our findings.

In conclusion, our findings underscore the potential risks associated with levornidazole and IMP-II, highlighting the need for further investigation and regulatory scrutiny to ensure patient safety. The methodological approach used in this study presents a strong framework for future research on drug toxicity and safety, thereby contributing to the advancement of drug quality control and medication safety.

## Figures and Tables

**Table 1:** Hepatotoxicity prediction model parameters.

Item	Basic Parameters		Item	Model Parameters		Item	Model Parameters	
**Random Seed**			**Number of Monte Carlo tries**			**Max Steps**		
Model Types	ANN	34,590	Model Types	ANN	1	Model Types	ANN	-
	SVM	65,600		SVM	-		SVM	30,000
**Percent**			**Early stopping count**			**Networks multiple**		
Model Types	ANN	10	Model Types	ANN	15	Model Types	ANN	5
	SVM	10		SVM	-		SVM	5
**Sensitivity Analysis**			**Mutation Rate**			**Ensemble Selection Method**		
Model Types	ANN	TLA	Model Types	ANN	-	Model Types	ANN	DP
	SVM	GA		SVM	0.9		SVM	DP
**Test Set Selection**			**Per ensemble**					
Model Types	ANN	KSOP	Model Types	ANN	33			
	SVM	KSOP		SVM	33			

TLA: a pseudo inverse MLR analysis to determine the characteristic of the descriptors; KSOP: kohonen self-organizing map, an unsupervised learning algorithm widely used in deep learning; GA: Genetic Algorithm, an optimization algorithm that simulates the Darwinian process of biological evolution; DP: Dot product, model complexity metric, considers the product of the test set metric with the geometric mean of the training and verification sets, autoscaled ([0.5, 1]); “-”:without involving.

**Table 2:** Performance of hepatotoxicity prediction model.

Model Type	NO	Ensemble Model		Performance								
		Neurons	Inputs	Sensitivity		Specificity		Youden		False Rate		Min Confidence
				Training	Test	Training	Test	Training	Test	Training	Test	Training
**SVM**	**1**	**-**	6	0.78	0.5	0.78	0.85	0.56	0.35	0.22	0.21	-
	2	-	28	0.82	0.75	0.81	0.85	0.63	0.6	0.18	0.17	-
	3	-	50	0.83	0.75	0.78	0.85	0.61	0.6	0.19	0.17	-
	4	-	116	0.83	0.75	0.79	0.82	0.62	0.57	0.19	0.19	-
	5	-	138	0.82	0.88	0.78	0.82	0.6	0.7	0.2	0.17	-
	mean	-	-	0.816	0.726	0.788	0.838	0.604	0.564	0.196	0.182	-
ANN	1	1	48	0.91	0.82	0.92	1	0.83	0.82	0.09	0.13	0.55
	2	1	64	0.94	0.86	0.94	0.5	0.88	0.36	0.06	0.23	0.59
	3	1	80	0.94	0.86	0.94	0.93	0.88	0.39	0.06	0.2	0.56
	4	1	112	0.92	0.86	0.92	0.75	0.81	0.61	0.08	0.17	0.50
	5	2	48	0.92	0.82	0.92	0.88	0.94	0.69	0.08	0.17	0.56
	mean	-	-	0.926	0.844	0.928	0.812	0.868	0.574	0.074	0.18	0.552

Youde index: also known as Youden statistic, a metric used to evaluate the performance of a binary classification model, the formula is as follows: Youden Index = Sensitivity + Specificity − 1, the value ranges from 0 to 1, with larger values indicating better performance of the classification model; False Rate: fraction of incorrect predictions, False Rate = (FN + FP)/(TP + FN + TN + FN), with smaller values indicating better performance of the classification model; Min Confidence: reflects the quality of the confidence model linked to each ensemble, serving as a complement to other classification quality metrics. A value below 0.5 typically indicates a split confidence model, while a value above 0.5 is indicative of a unified confidence model.

**Table 3:** System resulting data of standard experiment.

Structure	Identifier	ANN1
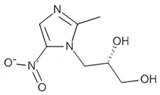	IMP-III	0 (54%)
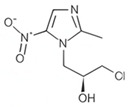	levornidazole	1 (99%)
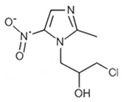	ornidazole	0 (99%)
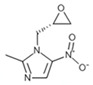	IMP-II	0 (56%)

**Table 4:** Online prediction results of potential hepatotoxicity of levornidazole and its main impurities.

Compound Name	Predictive Tool					
	eMolTox [26]	Vienna LiverTox [27]	LimTox [28]		ADMETLab [29]	
Type	Liver Injury	Liver Injury	Liver Injury	Autoimmune Hepatitis	Liver Injury	Hepatotoxicity
levornidazole	+/0.998	+/0.69	+/0.36	+/0.34	+/0.914	+/0.806
ornidazole	+/0.983	+/0.66	\	\	+/0.914	+/0.806
IMP-II	+/0.998	+/0.69	\	\	+/0.884	+/0.682
IMP-III	+/0.983	+/0.65	\	\	+/0.878	+/0.620

“+/0.998”: Positive/Possibility value, “\”: non-result.

## Data Availability

Data supporting these findings can be requested from the corresponding author.

## Supplementary Materials

The following supporting information can be downloaded at: https://www.mdpi.com/article/10.3390/molecules30050995/s1, Table S1: Mean of molecular descriptor. Table S2: Compound information table for modeling.
