# Supplementary material for: Hepatotoxicity Evaluation of Levornidazole and Its Three Main Impurities: Based on Structure–Toxicity Classification Prediction Combined with Zebrafish Toxicity Assessment
Source: Molecules. 2025 Feb 21;30(5):995. doi: 10.3390/molecules30050995 (PMC11901814; doi:10.3390/molecules30050995)
Supplement: Supplementary file 1 [file molecules-30-00995-s001.zip › molecules-3429913-supplementary.pdf]

Supplementary Table 1. Mean of molecular descriptor

Name	Category	Implication
EEM_NFnp	Charge-based Descriptors	Minimum sigma Fukui index on nonpolar atoms
Pi_FPl5		Fifth component of the autocorrelation vector of pi Fukui(+) indices
NPA_Q6		Sixth component of the autocorrelation vector of estimated NPA partial atomic charges
Pi_AQc		As above, but only on C atoms
EEM_XFpl		Maximum sigma Fukui index on polar atoms
Pi_FMi2		Second component of the autocorrelation vector of pi Fukui(-) indices
Sulfide_-S-	Functional Groups	Number of Sulfide groups
HBAoch	Hydrogen Bonding Descriptors	Sum of Estimated NPA Partial Atomic Charges on Oxygen-based HB Acceptors
QAvgNeg	Molecular Ionization Descriptors	Absolute Value of the Population Average Across All Ionized Species of the Net Formal Calculated at Specified pH (default = 7.4)Negative Charge
N_IoAcAt		Number of recognized ionizable atom types that are acidic
N_Pisyms	Simple Constitutional Descriptors	Number of distinct π systems, excluding lone pairs
N_Sulfur		Number of sulfurs

Supplementary Table 2. Compound information table for modeling

Canonical SMILES	Identifier	hepatoxicity	Canonical SMILES	Identifier	hepatoxicity
C(=O)(c1cc2c(Sc3c(N2CCCN4CCN(CCO)CC4)cccc3)cc1)C	100	1	N(CCN1C(=O)CN=C(c2c(F)cccc2)c3c1ccc(Cl)c3)(CC)CC	253	1
C(N(N=O)CCCl)(=O)NC1CCCCC1	1006	0	Fc1c(Cl)cc(Nc2c3c(ncn2)cnc(n3)NC4CCN(CC4)C)cc1	27	1
Clc1ccc(C2(c3c(C4=NCCN42)cccc3)O)cc1	1011	0	C(=O)(C1C(=O)c2c(nc(c(F)c2)N3C/C(=N\OC)/C(CN)C3)N(C=1)C4CC4)O	271	1
C(=O)(C1C2(C(C3C(C4(C(=CC(=O)CC4)C(C3)C)C)C(O)C2)CC1)C)C	1014	0	C(=N/N=C/c1c(Cl)cccc1Cl)(N)N	273	1
C(=O)(Nc1c(cccc1C)C)C2N(CCCC2)C	1018	0	C(N=C(N)N)(=O)Cc1c(Cl)cccc1Cl	274	1
c1(C(C(N)C)O)cc(O)ccc1	1020	0	C(F)(F)(F)c1cc2c3c(c(Cl)cc(Cl)c3)cc(c2cc1)C(O)CCN(CCCC)CCCC	275	1
C(=O)(OCC(O)COc1c(OC)cccc1)N	1024	0	O=C1C2(N=C(N1Cc3ccc(-c4c(-c5nn[nH]n5)cccc4)cc3)CCCC)CCCC2	289	1
O=C1C(c2ccccc2)(CC(=O)N1C)C	1026	0	C(=O)(c1ncc(Cl)cc1)NCCN	297	1
O=C1C2C34c5c(c(O)ccc5CC(C3(O)CC1)[N+](CC6CC6)(CC4)C)O2	1027	1	Clc1cc(C(c2cc3c(nc[nH]3)cc2)n4cncc4)ccc1	302	1
C(=O)(Oc1c(c(c(OCC(O)CNC(C)C)cc1C)C)C)C	1028	0	C(=O)(c1c2c(n(n1)Cc3c(Cl)cc(Cl)cc3)cccc2)O	305	1
O(c1ccc(O)cc1)Cc2ccccc2	1036	0	Clc1cc2C(=Nc3c(Oc2cc1)cccc3)N4CCN(CC4)C	307	1
C(c1cc2c(c(O)c1)C3C(C(O2)(C)C)CCC(=O)C3)(CCCCCC)(C)C	1039	1	C(=O)(OC(Cc1ccc(Cl)cc1)(C)C)C(N)C	31	1
c1(cnccc1)C2N(CCC2)C	1046	0	C(=O)(C(Cc1ccc(N(CCCl)CCCl)cc1)N)O	311	1
C(=O)(N1C(C(=O)NC(C(=O)O)Cc2ccccc2)CCC1)C(CSC(=O)C)C	105	1	C(=O)(c1c(O)ccc(c1)N)O	313	1
C(=N/OCc1c(Cl)cc(Cl)cc1)(\c2c(Cl)cc(Cl)cc2)/Cn3cncc3	1051	1	C(Cl)(Cl)(Cl)C(c1ccc(OC)cc1)c2ccc(OC)cc2	322	1
O(c1c(OCC(O)CNC(C)C)cccc1)CC=C	1052	0	O(c1ccc(cc1)CCOC)CC(O)CNC(C)C	328	1
C(c1c(c(c(c(c1)C)CC2=NCCN2)C)O)(C)(C)C	1053	1	C(=O)(O)CC/C(=C/Cc1c(OC)c(c2c(C(=O)OC2)c1O)C)/C	341	1

C(C(c1ccccc1)(O)C2CCCCC2)(=O)OCC3=NCCCN3C	1054	1	C(=O)(C(NC(=O)C1CCC(C(C)C)CC1)Cc2ccccc2)O	346	1
C(F)(F)(F)c1c(Cl)ccc(C2(O)CCN(CCCC(c3ccc(F)cc3)c4ccc(F)cc4)CC2)c1	1058	1	C(=O)(c1c(nccc1)Nc2cc(C(F)(F)F)ccc2)O	352	1
S(CC1CN(C2C(c3c4c(ccc3)[nH]cc4C2)C1)CCC)C	1061	1	N(/N1C(=O)NC(=O)C1)=C\c2oc([N+](=O)[O-])cc2	358	1
O=C1C(=C(Oc2c1cccc2)O)C(c3ccccc3)CC	1063	1	Clc1c(Cl)cccc1	363	1
O=C1OCC2C1C(c3c(cc4c(OCO4)c3)C2O)c5cc(OC)c(OC)c(OC)c5	1070	1	C(=O)(C1C(=O)C=C/C(=N\Nc2cc(C(=O)O)c(O)cc2)/C=1)O	365	1
C(=O)(OC1(C(=O)COC(=O)CC)C2(C(C3C(C4(C(=CC(=O)C=C4)CC3)C)C(O)C2)CC1)C)OCC	1075	1	N(=N/c1ccccc1)\c2ccc(N(C)C)cc2	379	1
C(=O)(C(NCCC)C)Nc1c(cccc1)C	1076	0	Clc1cc2c(Sc3c(N2CCCN4CCN(CCO)CC4)cccc3)cc1	388	1
C(=O)(OCCN(CC)CC)c1ccc(cc1)N	1077	0	C(=O)(NC(=O)N)Cc1ccccc1	389	1
C(=O)(OCCN(CC)CC)c1cc(c(OCCC)cc1)N	1079	1	C(=O)(C1CCN(CCCN2c3c(Sc4c2cccc4)ccc(Cl)c3)CC1)N	39	1
Fc1ccc(C(c2ccc(F)cc2)N3CCN(c4nc(nc(n4)NCC=C)NCC=C)CC3)cc1	108	1	C(=O)(O)Cc1c(-c2ccc(Cl)cc2)cn(-c3ccc(F)cc3)n1	397	1
C(#C)CNC1c2c(cccc2)CC1	1087	0	C(=O)(OCC(O)CO)c1c(Nc2c3c(ncc2)cc(Cl)cc3)cccc1	4	1
Clc1cc2C(c3ccccc3)=NCc4nnc(n4-c2cc1)C	109	1	C(=O)(c1ccc(cc1)N)NCCN(CC)CC	405	1
C(N(CC)C)(=O)Oc1cc(C(N(C)C)C)ccc1	1093	0	C(=O)(CCCN/C(/c1ccc(Cl)cc1)=C2/C(=O)C=CC(F)=C/2)N	406	1
N(CCc1c2c(ccc(c2)Cn3ncnc3)[nH]c1)(C)C	1094	1	O=S(=O)(c1c(Cl)cc2c(C(=O)NC(N2)CC)c1)N	414	1
N(CCc1c2c(ccc1)NC(=O)C2)(CCC)CCC	1095	1	c1(C(C(NCCc2ccc(O)cc2)C)O)ccc(O)cc1	418	1
N(C1Cc2c(c(O)ccc2)CC1)(CCc3sccc3)CCC	1097	1	C(=O)(O)Cc1cc(Cl)c(OCC=C)cc1	42	1

C(c1cc2c(OC(O2)C)cc1)(C)(C)C	1099	1	O(C(c1c(Cl)cc(Cl)cc1)Cn2cncc2)Cc3c4c(c(Cl)ccc4)sc3	424	1
C(=O)(O)Cc1nc(-c2ccc(Cl)cc2)sc1	11	1	O=S(=O)(c1ccc(cc1)N)Nc2c(c(no2)C)C	436	1
C(=O)(NS(=O)(=O)c1ccc(cc1)N)C	1108	1	C(#CC1CC1)C2(C(F)(F)F)c3c(ccc(Cl)c3)NC(=O)O2	44	1
O(C(c1c(Cl)cc(Cl)cc1)Cn2cncc2)Cc3c(Cl)scc3	1121	1	N(CC(C(c1cc(O)ccc1)CC)C)(C)C	440	1
C(=O)(O)COc1c2c(ccc1)CC3C(C(O)CC3C2)CCC(O)CCCCC	1127	0	C(=O)(c1c(n(C(=O)N)c2c1cc(Cl)cc2)O)c3sccc3	443	1
C(=O)(Nc1ccc(/C=N/NC(=S)N)cc1)C	113	1	O=C1c2c(C(=O)N1C3C(=O)NC(=O)CC3)cccc2	449	1
C(F)(F)(F)C1C(=O)NC(=O)N(C=1)C2OC(C(O)C2)CO	1130	1	C(=O)(c1sc(C(C(=O)O)C)cc1)c2ccccc2	454	1
C(N(Cc1ccncc1)CC)(=O)C(c2ccccc2)CO	1133	0	C1(=NCCN1)Cc2ccccc2	459	1
O1C(O)C(O)C(O)C(O)C1	1143	0	C(=C(/c1ccccc1)\CCCl)(\c2ccc(OCCN(C)C)cc2)/c3ccccc3	461	1
N(CCc1c2c(ccc(c2)CC3NC(=O)OC3)[nH]c1)(C)C	1146	0	C(=O)(c1c(NC(=O)/C=C/c2cc(OC)c(OC)cc2)cccc1)O	463	1
O(c1ccc(cc1)CCOCC2CC2)CC(O)CNC(C)C	1150	1	Clc1c(C2c3c(-n4c(nnc4C)CN=2)ccc(Cl)c3)cccc1	464	1
C(=O)(C(NC(=O)C)COC)NCc1ccccc1	1163	0	O(c1ccccc1)CC(NN)C	48	1
C(NCC(O)COc1c2c(C(=O)CCC2)ccc1)(C)(C)C	1164	0	ClCCc1c(ncs1)C	491	1
O=S(=O)(c1c(Cl)cc2c(S(=O)(=O)N(C(N2)CCl)C)c1)N	1168	1	C(C(=C)CC)(=O)c1c(Cl)c(Cl)c(OCC(=O)O)cc1	497	1
C(=O)(c1cnn(-c2nc3c(c(n2)N)ncn3C4OC(C(O)C4O)CO)c1)NC	1177	0	C(=O)(c1c(-c2c(F)cccc2Cl)noc1C)NC3C(=O)N4C(C(=O)O)C(SC43)(C)C	499	1
O=C1N(c2c(C(c3ccccc3)=NC1O)cc(Cl)cc2)C	1181	1	C(=O)(c1ccncc1)NNC(C)C	5	1
O(c1ccc(N2CCN(C(C)C)CC2)cc1)CC3OC(c4c(Cl)cc(Cl)cc4)(OC3)Cn5ncnc5	1182	1	C(=O)(O)CCCCCC(C1C(=O)C(=C(C(=O)C=1C)C)C)c2ccccc2	517	1
C(=O)(Nc1c(cccc1C)C)C2N(CCCC)CCCC2	1191	0	C(=O)(c1sccc1)c2ccc(C(C(=O)O)C)cc2	522	1
N(CCOC(c1ncccc1)c2ccc(Cl)cc2)(C)C	1192	0	C(=O)(Oc1ccc(C(c2ccc(OC(=O)C)cc2)=C3CCCCC3)cc1)C	533	1

N(CCOC(c1ccccc1)c2ccccc2)(C)C	1196	0	C(=O)(Oc1c(cc(OCCN(C)C)c(C(C)C)c1)C)C	538	1
O(c1c(OC)cccc1)CC(O)CO	1201	0	S(c1c(c(Cl)cc(Cl)c1)O)c2c(c(Cl)cc(Cl)c2)O	54	1
O(CCN1CCN(C(c2ccc(Cl)cc2)c3ccccc3)CC1)CCO	1203	1	Clc1c(cccc1)CN2Cc3c(scc3)CC2	544	1
Clc1ccc(C(c2ccccc2)N3CCN(Cc4cc(ccc4)C)CC3)cc1	1205	1	C(=O)(O)Cc1ccc(cc1)CC(C)C	550	1
Fc1c(C2c3c(-n4c(ncc4CN=2)C)ccc(Cl)c3)cccc1	1209	1	C(=O)(c1c2c(n(c1C)CC(=O)O)cc(OC)cc2)c3ccc(Cl)cc3	552	1
N(C(COc1ccccc1)C)(Cc2ccccc2)CCCl	1212	1	C(=O)(O)Cc1c(Nc2c(F)cccc2Cl)ccc(c1)C	560	1
C(=O)(OCC1C2C(=O)C(=C(C(=O)C=2N3C1(OC)C4C(N4)C3)C)N)N	1221	0	C(=O)(Nc1ccc(O)cc1)C	579	1
C(=O)(NC1C(=O)N2C(C(=O)O)C(SC21)(C)C)Cc3ccccc3	1224	1	O=C1C(c2ccc(cc2)N)(CC)CCC(=O)N1	581	1
O=C1C(=O)C(=C(c2c1cccc2)O)C3CCC(c4ccc(Cl)cc4)CC3	123	1	O=C1N(C(c2ccc(Cl)cc2)S(=O)(=O)CC1)C	584	1
c1(C(C(NC)C)O)ccccc1	1230	0	O=C1Oc2c(cc(Cl)cc2)N1	585	1
c1(ccccc1)CC(NC)C	1232	0	N(CCCN1c2c(ccc(Cl)c2)CCc3c1cccc3)(C)C	586	1
N(CCC(c1ncccc1)c2ccc(Cl)cc2)(C)C	1237	0	C(=O)(O)Cc1c(Nc2c(Cl)cccc2Cl)cccc1	587	1
Clc1nc(nc(n1)NCC)NC(C)C	124	1	C(=O)(c1c(O)ccc(-c2c(F)cc(F)cc2)c1)O	588	1
N(C(C)C)(C(C)C)CCC(c1c(O)ccc(c1)C)c2ccccc2	1242	0	C(=O)(c1noc(c1)C)NNCc2ccccc2	597	1
O=CNc1c(O)ccc(C(O)CNC(Cc2ccc(OC)cc2)C)c1	1253	0	C(=O)(c1c(O)ccc(C(O)CNC(CCc2ccccc2)C)c1)N	600	1
C(=O)(O)CC(c1ccc(Cl)cc1)CN	126	1	C(=O)(C(c1cc(Cl)c(N2CC=CC2)cc1)C)O	619	1
C(NCC(c1cc(c(O)cc1)CO)O)(C)(C)C	1263	0	C(=O)(c1c(Cl)c(Cl)c(OCC(=O)O)cc1)c2sccc2	620	1
O=C1C(c2c(Cl)cccc2)(NC)CCCC1	1275	1	C(C(Cc1cc(c(O)cc1)O)(N)C)(=O)O	621	1
C(C(c1ccccc1)(c2ccccc2)CCN3CCC(c4ccc(Cl)cc4)(O)CC3)(N(C)C)=O	1276	0	C(=O)(C(c1cc2c(oc(-c3ccc(Cl)cc3)n2)cc1)C)O	622	1

C(=O)(NCC(c1c(OC)ccc(OC)c1)O)CN	1280	0	C(N(CCC)CCC)(=O)Cc1c(-c2ccc(Cl)cc2)nc3n1C=C(Cl)C=C3	628	1
C1(N=C(N2CCCCC2)C=C(N1O)N)=N	1281	1	N(C1C(=O)NC(=O)NC=1)(CCCl)CCCl	63	1
Clc1c(C(c2ccc(Cl)cc2)C(Cl)Cl)cccc1	1282	1	O(c1c2c(ccc1)cccc2)C(c3sccc3)CCNC	633	1
C(N(C)C)(=O)Oc1c[n+](ccc1)C	1287	0	C(=O)(NS(=O)(=O)c1ccc(C(=O)C)cc1)NC2CCCCC2	653	1
Clc1ccc(-c2c(nc(nc2N)N)CC)cc1	1288	1	N(Cc1c(O)ccc(Nc2c3c(ncc2)cc(Cl)cc3)c1)(CC)CC	656	1
C(=O)(OCCN(C)C)c1ccc(NCCCC)cc1	1291	0	C(=O)(NS(=O)(=O)c1ccc(Cl)cc1)NCCC	664	1
N(c1cc(O)ccc1)(c2ccc(cc2)C)CC3=NCCN3	1292	0	C(C(Oc1ccc(cc1)C2C(Cl)(Cl)C2)(C)C)(=O)O	665	1
C(#C)CN(C(Cc1ccccc1)C)C	1308	0	C(C(Oc1ccc(Cl)cc1)(C)C)(=O)OCC	666	1
C(OCCC1N(CCC1)C)(c2ccc(Cl)cc2)(c3ccccc3)C	1309	0	Clc1c(c(Cl)ccc1)NC2=NCCN2	667	1
c1(c(O)ccc(c1)CCNC(CCc2ccc(O)cc2)C)O	1310	0	Clc1cc2c(N=C3C(=C(N4CCN(CC4)C)N2)C=CC=C3)cc1	668	1
C(=O)(c1ccc(cc1)N)O	1331	1	C(C(Oc1ccc(C(=O)c2ccc(Cl)cc2)cc1)(C)C)(=O)OC(C)C	676	1
C(=O)(Cc1ccc(OCC(O)CNC(C)C)cc1)N	1334	0	Clc1c(Cl)cc(Cl)c(c1Cc2c(Cl)c(Cl)cc(Cl)c2O)O	685	1
c1(c(C(C)C)cccc1C(C)C)O	1355	0	O=S(=O)(c1c(Cl)cc2c(S(=O)(=O)NCN2)c1)N	687	1
C(=O)(c1c(cc(n1C)CC(=O)O)C)c2ccc(Cl)cc2	1359	1	O=P1(N(CCCl)CCCO1)NCCCl	688	1
C(C(Oc1ccc(cc1)CCNC(=O)c2ccc(Cl)cc2)(C)C)(=O)O	1375	1	C(=O)(c1ccc(Cl)cc1)n2c3c(c(c2C)CC(=O)O)cc(OC)cc3	691	1
C(=O)(OCc1cc(Oc2ccccc2)ccc1)C3C(C3C=C(Cl)Cl)(C)C	1377	1	C(=O)(c1c(Nc2c(Cl)c(ccc2Cl)C)ccc c1)O	696	1
S(c1c(Cl)cccc1Cl)C(Cn2cncc2)CCc3ccc(Cl)cc3	1380	1	C(=O)(c1c(Nc2c(c(ccc2)C)C)cccc1)O	697	1
C(=O)(C(C1ON=C(Cl)C1)N)O	1388	1	C(=O)(c1c(OC)cc(c(Cl)c1)N)NCCN(CC)CC	700	1
C(SN1C(=O)C2C(C1=O)CC=CC2)(Cl)(Cl)C(Cl)Cl	139	1	O=S(=O)(c1c(Cl)cc2c(C(=O)N(c3c(cccc3)C)C(N2)C)c1)N	701	1

C(=O)(c1cc(Cl)ccc1)C(NC(C)(C)C)C	1392	1	C(=O)(c1c(O)cc(cc1)N)O	708	1
C(=O)(N1CCN(c2ccc(OCC3OC(c4c(Cl)cc(Cl)cc4)(OC3)Cn5cncc5)cc2)CC1)C	14	1	C(=O)(Nc1ccc(OCC)cc1)C	709	1
C(=O)(C(C(=O)O)c1ccccc1)NC2C(=O)N3C(C(=O)O)C(SC32)(C)C	140	1	C(=O)(O)COc1c(Cl)c(Cl)c2C(=O)C(c3ccccc3)(Cc2c1)C	71	1
C(=O)(c1nn(cn1)C2OC(C(O)C2O)CO)N	1408	0	C(=O)(c1ccc(cc1)CNNC)NC(C)C	711	1
C(=O)(OCC)N1C(=S)N(C=C1)C	141	1	O=C1N2C(=NN1CCCN3CCN(c4cc(Cl)ccc4)CC3)C=CC=C2	719	1
Clc1c(Cl)ccc(c1)C2c3c(cccc3)C(NC)CC2	1415	0	C(=O)(Nc1cc2c(-c3c(cccc3)C2)cc1)C	728	1
C(=O)(NS(=O)(=O)c1ccc(cc1)N)NCCCC	142	1	C(=O)(c1c(-c2c(Cl)cccc2)noc1C)NC3C(=O)N4C(C(=O)O)C(SC43)(C)C	734	1
C(=O)(C1=C(Cl)CSC2N1C(=O)C2NC(=O)C(c3ccccc3)N)O	146	1	C(=O)(NC(C(Cl)C)C1OC(SC)C(O)C(O)C1O)C2N(CC(CCC)C2)C	743	1
C(=O)(C1=C(CSc2nnc(s2)C)CSC3N1C(=O)C3NC(=O)Cn4nnnc4)O	147	1	C(=O)(C1C(=O)c2c(N(C=1)C3CC3)cc(c(F)c2C)N4CC(NCC4)C)O	753	1
C(/C(=N/OC)/c1nc(sc1)N)(=O)NC2C(=O)N3C(C(=O)O)=C(COC(=O)C)CSC32	156	1	O=C1c2c(OC)cc(OC)c(Cl)c2OC13C(OC)=CC(=O)CC3C	756	1
C(N(C(c1sc2c(c1)cccc2)C)O)(=O)N	16	1	C(=C(/c1ccc(OCCN(CC)CC)cc1)\c2ccccc2)(/Cl)\c3ccccc3	762	1
C(/C(/c1nc(sc1)N)=C/CC(=O)O)(=O)NC2C(=O)N3C(C(=O)O)=CCSC32	161	1	C(=O)(C(c1ccc(cc1)CC(C)C)C)O	776	1
C(/C(=N/OC)/c1occc1)(=O)NC2C(=O)N3C(C(=O)O)=C(COC(=O)N)CSC32	163	1	C(=O)(O)COCCN1CCN(C(c2ccc(Cl)cc2)c3ccccc3)CC1	777	1
C(=O)(O)CCCc1ccc(N(CCCl)CCCl)cc1	166	1	C(=O)(C(Cl)Cl)NC(C(c1ccc([N+](=O)[O-])cc1)O)CO	778	1
C(=O)(C(Cl)Cl)NC(C(c1ccc([N+](=O)[O-])cc1)O)CO	167	1	C(=O)(c1c(O)cccc1)O	779	1
S(c1ccc(Cl)cc1)Cc2ccc(Cl)cc2	168	1	c1(c(c(cnc1C)CO)CO)O	781	0
C(=O)(OC)C(c1c(Cl)cccc1)N2Cc3c(scc3)CC2	180	1	c1(c([n+](cs1)Cc2c(nc(nc2)C)N)C)CCO	782	0
O=[N+](c1nc(n(-c2ccc([N+](=O)[O-])cc2)c1)C)[O-]	19	1	C(=O)(OCCOCCN(CC)CC)C1(c2ccccc2)CCCC1	783	0

O=C1N=C(N=CN1C2OC(C(O)C2)CO)N	190	1	Clc1ccc(C(c2ccccc2)N3CCN(CC3)C)cc1	784	0
Clc1cc2c(C(c3c(cccn3)CC2)=C4CCNCC4)cc1	195	1	C(=O)(Oc1ccc(C(c2ncccc2)c3ccc(OC(=O)C)cc3)cc1)C	810	0
C(F)(F)(F)c1cc(ccc1)CC(NCC)C	196	1	N(CCOC(c1ccccc1)c2ccccc2)(C)C	822	0
O=C1NC(=O)CN(C(CN2CC(=O)NC(=O)C2)C)C1	198	1	O=C1c2c(C(=O)C1c3ccc(OC)cc3)cccc2	865	1
O=S1(=O)c2c(ccc(Cl)c2)NC(=N1)C	202	1	Clc1c(c(Cl)cc(c1)N)NC2=NCCN2	868	1
C(=C(/c1ccc(O)cc1)\CC)(\c2ccc(O)cc2)/CC	203	1	O=C1c2c(C(=NN1C3CCN(CCC3)C)Cc4ccc(Cl)cc4)cccc2	871	0
C(=O)(c1ccc(-c2ccccc2)cc1)C(OCC)Nc3ccc(C(=O)O)cc3	21	1	C(=O)(O)CCCc1nc2c(n1C)ccc(N(CCCl)CCCl)c2	874	1
O=S(=O)(OCCOc1c(Cl)cc(Cl)cc1)O	210	1	Clc1c(c2c(c(Cl)c1)cccn2)O	900	1
C(=C(/c1ccccc1)\CC)(\c2cc(O)ccc2)/c3ccc(OCCN(C)C)cc3	215	1	O=S(=O)(c1c(Cl)ccc(C2(c3c(C(=O)N2)cccc3)O)c1)N	902	0
C(=O)(OCC)C(NC(C(=O)N1C(C(=O)O)CCC1)C)CCc2ccccc2	219	1	C(C(Oc1ccc(C(=O)c2ccc(Cl)cc2)cc1)(C)C)(=O)NCCS(=O)(=O)O	903	0
C(=O)(C1=C(NC(=O)N1)C)c2ccc(SC)cc2	222	1	C(N(c1c(cccc1)C)CC)(=O)/C=C/C	912	0
C(/C(/C(N(CC)CC)=O)=C\c1cc(c(c(O)c1)O)[N+](=O)[O-])#N	223	1	C(=O)(OCCN(C)C)C(c1ccccc1)C2(O)CCCC2	915	0
C(N(CCCl)CCCl)(=O)Oc1cc2c(cc1)C3C(C4C(C(O)CC4)(CC3)C)CC2	228	1	C(=O)(C(N(CCC)CC)CC)Nc1c(cccc1C)C	954	0
C(#N)c1cc(c(Oc2c(Br)c(nc(n2)Nc3ccc(C#N)cc3)N)c(c1)C)C	240	1	C(=O)(OCC)c1n(C(c2ccccc2)C)cnc1	955	0
C(=O)(c1ccc(-c2ccccc2)cc1)CCC(=O)O	244	1	C(=O)(O)Cc1c(-c2ccc(Cl)cc2)nc(-c3ccccc3)s1	247	1
Clc1c(c(O)cc2c1CCNCC2c3ccc(O)cc3)O	245	1			

*: training, test set division number; **: hepatotoxicity classification, "1" toxicity, "0" non-toxicity
